# Relationship Between Neurodevelopmental Areas and Difficulties in Emotional-Behavioural Variables in Children With Typical Development Under 2 Years of Age: Sex Differences

**DOI:** 10.5334/pb.1203

**Published:** 2024-09-03

**Authors:** Maravillas Castro, Visitación Fernández, Antonia Martínez, Mavi Alcántara, Almudena Campillo, Concepción López-Soler

**Affiliations:** 1 Faculty of Psychology, University of Mrucia, 30100 Murcia, Spain

**Keywords:** Bayley-III scale, CBCL 1.5–5 scale, neurodevelopment, emotional/behavioural problems, sex difference, toddlers

## Abstract

The aim of this study was to examine the relationship between neurodevelopmental areas and possible difficulties in emotional-behavioural variables, and to determine if sex moderated this relationship. A community sample of 231 boys and girls with typical development and with a mean age of 19.84 months was evaluated, using the Bayley-III and CBCL 1.5–5 scales. The main results confirmed: (1) better linguistic abilities in girls in both language areas (receptive communication and expressive communication), finding more evidence according to the Bayesian analysis in expressive communication; (2) in the emotional-behavioural area girls had higher scores in withdrawal; (3) significant negative correlations of low magnitude were found between the Bayley and CBCL scales, particularly in the areas of language and cognitive and internalising and externalising problems; (4) children with low cognitive abilities and those with poor receptive communication showed more inter and externalising difficulties; (5) no significant predictive value or moderating effect of sex was found, (6) the number of participants who simultaneously manifested significant deficits in both domains (neurodevelopmental and emotional-behavioural) was very reduced. Future research should corroborate these results and the characteristics of the relationship found at these early ages. Detecting the population at risk in the first two years of life would enable the implementation of interventions aimed at improving neurodevelopmental deficits and emotional-behavioural problems. Thus, identification of deficits in one domain should lead to evaluation of the other.

## Introduction

Child development describes the processes of change and stability incorporating physical (e.g., motor), cognitive (e.g., intellectual and language), and socioemotional growth (Tamayo et al., 2023). Evidence suggests these domains influence one other and mainly follow a stable trajectory, tending to develop at the same time (Frazier, 2011). During the first three years of life, the speed of development is rapid and important changes take place in cognitive, language, and social skills (Brito et al., 2019).

In this line, the neuro-constructivist conception states that genetic and socioenvironmental conditions vary in magnitude and time, and can produce different developmental trajectories, typical or atypical. Thus, the existence of a disorder in neurodevelopmental areas would imply genetic mutations and also the presence of variables that modify the environment where the child develops (Defagó, 2012; Karmiloff-Smith et al., 2012; Soukup-Ascençao et al., 2016). Therefore, it is relevant to study which aspects of specificity of different domains immersed in dynamic social contexts can constitute the center of typical and atypical development of human cognition. Young children also show great variability within and between individuals, which might reflect the emergent differentiation of functional systems (Karmiloff-Smith et al., 2012).

At present, the Bayley Scales of Infant and Toddler Development (BSID-II, Bayley, 1993; Bayley-III. Bayley, 2006), and CBCL (Child Behavior Checklist) (Achenbach & Edelbrock, 2000) are widely used for assessment of neurodevelopmental and emotional-behavioural difficulties in young children. The Bayley-III (Bayley, 2006; CDIAP Parc Taulí, University of Murcia and the Pearson Clinical & Talent Assessment R&D Departament, 2015), is broadly used in clinical and research settings, especially to identify neurodevelopmental disabilities. Identification of possible delays would not lessen long term effects, but the Bayley test can be used for intervention planning, which in turn could possibly lessen long term effects (Krogh & Vaever, 2019; Lung et al., 2009). In addition, it provides information on the child’s skills and strengths. The Child Behavior Checklist (CBCL 1.5–5 years) belongs to the multiaxial classification system proposed by Achenbach & Rescorla (2000), whose purpose is the evaluation of emotional and behavioural problems in children aged between a year and a half and five years. The psychometric tests report elevated reliability coefficients and good validity, since it allows discriminating children with and without emotional and behavioural problems (Achenbach & Rescorla, 2000).

### Influence of sex in development

Knowing the influence of sex as a risk and/or protection factor in the early stages of child development is a priority. Determining the magnitude of these differences in the domains of development helps to design more specific prevention and intervention programs. The literature has identified male sex as a risk factor for early development of neurodevelopmental and emotional/behavioural disorders (McCarthy, 2016). The explanatory factors for sex differences are complex, as they include interactions between biological factors (genetic and hormonal, among others), exposure to environmental factors (such as stress) and sociocultural factors. There is great scientific interest in this field, however, current findings do not yet allow us to determine the specific mechanisms that provide clarity regarding these differences (May et al., 2019; Merikangas & Almasy, 2020). Below are several studies that reveal differences in development between boys and girls.

In the cognitive area, girls have performed better than boys. In studies with samples aged up to 36 months, through the Bayley scale, effect sizes were found from small, at 14 and 18 months (Koutra et al., 2012; Suhonen et al., 2018), to large, at 24 and 36 months (Krog & Væver, 2019). Lung et al. (2009) also reported sex as a significant predictor in relation to cognitive domain at 36 months.

As for area of language, the previous trend at early ages is also detected, generally being observed higher scores in girls, for example, Lung et al. (2009) in a sample of 36 month-old children. As regards receptive language, effect sizes range from small (eg, Henrichs et al., 2013; Koutra et al., 2012; Rautakoski et al., 2021; Suhonen et al., 2018) to medium (Krog & Væver, 2019); while in expressive language only some authors found significant differences, with small effect sizes (Henrichs et al., 2013; Koutra et al., 2012; Rautakoski et al., 2021; Suhonen et al., 2018). However, a recent review (Rinaldi et al., 2023) shows that the advantage of girls in early language acquisition is controversial, given no evidence has been found in any age group studied (from 8 to 83 months) and if so, it is slight.

As regards assessment of the motor area with the Bayley-III, Lung (2009) found that sex was a predictor at 36 months. Girls generally perform better in fine motor at 18 months, with a small effect size (Koutra et al., 2012), and at 7 and 36 months, with medium to large effect sizes (Krog & Væver, 2019), while these differences are not reflected in gross motor. Although it is typical to find a pattern with advantages for boys in gross motor skills and girls in fine motor skills, results are controversial in older samples (Giagazoglou et al., 2011; Kokštejn et al., 2017).

In the emotional-behavioural area, studies using the CBCL scale (Achenbach & Rescorla, 2000, 2001) find sex differences in pre-schoolers, with higher prevalence rate of problems in this area in boys (Rescorla et al., 2012). Furthermore, in preschool and school samples, it is stated that internalising problems are more frequent in girls (Marković et al., 2016; Pourhoussein et al., 2015; Shaikh & Shinde, 2018) and externalising problems in boys (Carneiro et al., 2016; Marković et al., 2016; Shaikh & Shinde, 2018). Research has also been published on younger children. Henrichs et al. (2013) evaluated children aged 18 and 36 months through the CBCL for 1.5–5 years. Results reveal a predominance of externalising problems in children at both ages. At 18 months there were no differences by sex, though at 36 months boys reached higher scores in externalising and internalising problems according to information provided only by the father; while the mother indicated said differences only in externalising problems. Nevertheless, Rautakoski et al. (2021) found no sex differences in total socio-emotional problems in 17-month-old children.

Overall, sex plays an important role in early childhood development, with male sex being a risk factor for neurodevelopmental and emotional-behavioral disorders. Current findings do not provide clear mechanisms for these sex differences.

### Relationship between development areas

Evidence suggests that internalising and externalising symptoms and areas of child neurodevelopment mainly tend to occur at the same time (Tamayo et al., 2023). It is necessary to research the characteristics of this relationship in the first years of life. Over two decades ago, research corroborated the relationship between the neurodevelopmental and emotional areas in children aged between 30 and 39 months. Children with difficulties in any of the areas assessed with the BSID-II scored higher on the CBCL scales 1.5–5 years withdrawal, attention problems, internalising problems and total problems, according to information provided by both parents. Moreover, boys with developmental deficits scored higher on all scales except mother-reported sleep problems. Parents also observed more problems in emotional reactivity, aggressiveness, and externalizing problems in boys in this group (Baker et al., 2002). Other studies have focused on examining the relationship between each neurodevelopmental area with the emotional-behavioural area, as described below.

#### Cognitive area and emotional problems

The cognitive area and emotional-behavioural problems are interrelated variables, though it is not yet known how they develop together in the general child population. In older populations, a small negative sign of statistically significant relationship has been found between cognitive ability and inter-and externalizing problems in pre-schoolers (Flouri et al., 2018; Morin et al., 2017). Co-developmental trajectories and their outcomes in adolescents have also been researched (Flouri et al., 2018; Racz et al., 2017).

#### Area of language and emotional problems

Regarding the area of language, various reviews and meta-analyses performed on studies of school samples (Chow & Wehby, 2018; Chow et al., 2018) confirmed a small negative relationship between early language skills and emotional-behavioural problems, both concurrent and posterior. This pattern of simultaneous development starts at a very young age, although data are limited and results inconsistent in children under 3 years of age (Manning et al., 2019; Roberts et al., 2018; Thurm et al., 2018). There is no consensus on whether the relationship differs by language domain (receptive-expressive) or emotional-behavioural area domain (internalising-externalising problems), and whether it exists across the entire distribution of language skills or only at the extremes (Chow et al., 2018; Clegg et al., 2015; Conway et al., 2017).

With respect to studies evaluating general language skills, Roberts et al. (2018) found in children between 18 and 36 months that there is less disruptive behaviour if language skills were more optimal, the association being stronger in girls. Thurm et al. (2018) compared a group of children with delays in both language areas (receptive and expressive) with another group that presented typical development. Results showed the former obtained higher scores in both internalising and externalising behaviours, with large effect sizes. Rautakoski et al. (2021) found that low level of receptive and expressive language did not predict emotional/behavioural problems at 17 months but did predict delays in socio-emotional skills.

In recent years, numerous investigations have been carried out relating expressive language evaluated at early ages (normally between 12 and 38 months), with behavioural problems. Some have only found a relationship with internalising type syndromes. For example, Henrichs et al. (2013) found an association between lower expressive vocabulary ability and risk of internalising problems at 18 months (36% risk; OR = 1.36, 95% CI [1.10, 1.69], *p* = .005), and also at 36 months for boys only (31% risk; OR = 1.31, 95% CI [1.04, 1.65], *p* = .02), based on information provided by the mother. The risk found by Conway et al. (2017) in their study (24–48 months) was 20%. Other research only found a relationship with externalising type problems. Thurm et al. (2018) indicated an association of expressive language at 18 months with externalising behaviours at 24 months. Manning et al. (2019) reported that children with fewer words spoken had more severe tantrums. Work has also been published revealing an association of expressive language with behavioural problems of both types, inter and externalising. Whitehouse et al. (2011) found the group of 24 month-old children with delayed speech achieved higher scores on inter and externalizing problems versus the control group, with small and null effect sizes, respectively. In the study by Morgan et al. (2015), 24 month-old children with a larger oral vocabulary presented fewer internalising and externalising behaviour problems at 60 months.

Receptive language has been included in a smaller number of works that relate language to the emotional-behavioural area. Henrichs et al. (2013) showed that receptive language at 18 months predicted externalising behaviour at 36 months, with a 44% risk, based on information provided by the father. Thurm et al. (2018) found an association of receptive language at 18 months with both inter and externalising behaviours at 24 months. And finally, Conway et al. (2017) indicate that receptive language had a negative linear association with internalising behaviours (*R*^2^ = 3.6%), and a curved negative association with externalising behaviours (*R*^2^ = 4.2%) only for those children who scored very low or very high in receptive language.

#### Motor area and emotional problems

Regarding the motor area, Castillejos and Ribera (2009) found a significant association between an aspect of gross motor skills (sensory processing behaviour) with the emotional/social area. Furthermore, the fine motor/perceptive area was also significantly related to the emotional/social area, in a sample of 3-year-old children. In work by Dowling et al. (2015), with children aged between 44 and 80 months, it was found that those at risk of presenting motor difficulties, scored significantly higher in total problems, externalising behaviour, aggressiveness and withdrawal, with small effect sizes. For their part, Piek et al. (2008) reported a statistically significant relationship with a negative sign between neuromuscular development and levels of anxiety/depression and withdrawal, reported by parents, in children between the ages of 3 and 5 years.

Generally, the cognitive area and emotional-behavioral problem are interrelated variables, however, it is not known in the general child population. The language area and the emotional-behavioral area have a small negative relationship between early skills and emotional-behavioral problems, whereas the motor area is signficantly relatd to emotional/social areas.

### Comorbidity of deficits in the areas of neurodevelopment and emotional problems

Various research has reported on prevalence rates of neurocognitive alterations and emotional problems in samples of young children. Some studies examine the community population in samples of boys from non-Western countries, using the Bayley scale with cut-off points adapted to said population. Cromwell et al. (2014) found in Malawian children the following prevalence rates of mild or severe delays in areas evaluated: cognitive (16.3%), expressive communication (15.9%), receptive communication (13.8%), gross (14.6%) and fine (16.1%) motor skills. For their part, Ballot et al. (2017) indicated 25.6% of children presented cognitive delay, 16.2% language delay, and 5.2% motor delay. Prevalence rates were much higher than those according to Western scales. Other studies included population at risk. Corral-Guillé and Rivera-González (2023) in infants and pre-schoolers from 0 to 42 months with a history of perinatal risk who presented developmental deficits found that 3.9% of the sample obtained low scores in the cognitive area, 8.5% in language, and 5.4% in the motor area. Yi et al. (2018) studied a sample with suspected developmental delay (mean age 16.6 months). These were evaluated with the BSID-II and the Bayley-III, with results showing that according to the Bayley-III cut-off point, 56.5% had delayed cognitive and language development, and 87.1% had motor development problems.

It is well documented that children even as young as 1.5 years, show internalising and externalising symptoms at an early age, as assessed using the CBCL, with considerable variation in symptoms (Achenbach et al., 2003). However, analysing these problems at such an early age can be challenging, as certain behaviours and emotions are also part of typical development (Tamayo et al., 2023). Most research includes samples of pre-schoolers. In the US population, prevalence rates in the clinical range were around 10% (Achenbach & Rescorla, 2010). In other works, it was in a very wide range, between 2.9% and 25.4% (Marković et al., 2016; Wu et al., 2012). Nonetheless, in samples under 36 months studies are scarcer. Results of the study by Pourhossein et al. (2015) confirmed the following prevalence rates of emotional-behavioural problems in children between the ages of 1.5 and 5 years: emotional reactivity (3.9%), anxiety/depression (6.8%), withdrawal (5.4%), somatic complaints (9.4%), sleep problems (6.2%), attention problems (5.7%), and aggressive behaviour (3.9%). When comparing age groups (1.5–5 vs 5–7 years), no statistically significant differences were observed. In contrast, Basten et al. (2016) examined problem behaviour profiles at ages 1.5 and 3 years, using latent profile analysis. Four similar profiles were found in both ages. In children of 1.5 years, 81.8% did not present problems (profile A); 11.1% had moderate externalizing problems, emotionally reactive behaviours, and low levels of anxiety-depression (profile B, externalising/emotionally reactive); 5.4% presented mild problems in all syndromes (profile C); and 1.7% showed both inter and externalising problems (profile D). In the 3-year-old group, a larger number of children were found without problems (86.5%, profile A); 6.5% of the sample was located in profile B; 4.8% in profile C (mild internalising), with scores only slightly higher in internalising than in externalising; and, finally, 2.2% presented profile D.

There are few community population studies reporting data on the prevalence in young children who concurrently present deficits in the areas of neurodevelopment (cognitive, language, and motor) and emotional-behavioural, using the Bayley and CBCL scales. To the best of our knowledge, the only study with these characteristics is that by Baker et al. (2002). They compared two groups (with and without deficits in some area of neurodevelopment) between 30 and 39 months, regarding the emotional area, evaluated by both parents. Results indicated that the group with developmental deficits had more significant emotional-behavioural problems than the normative group. According to information provided by mothers, the ratio was 1:3.1 (26.1% vs 8.3%), and by fathers 1:4.3 (24% vs 5.6%). Other studies use different scales to assess the relationship between specific domains of development, such as the work by Thurm et al. (2018). The coexistence of language and emotional problems was examined in two samples, one with expressive and receptive language delay and the other with typical development in this area. They also measured emotional/behavioural disturbances. In 18-month-old children, it was found that 20% of children with language delays presented important externalizing behaviours versus 2% of the normative group. The same proportion (20% vs 2%) was found for internalising type problems. At the age of 24 months, 15% of children with problems in both language areas (receptive and expressive) presented severe externalising behaviours, compared to 2% of the group with typical development. The proportion found in externalising problems was 8% for children with language difficulties, compared to 2% with normalised development. As described throughout this section, research on neurocognitive alterations and emotional problems in young children has shown variable prevalence rates. Based on review of the literature, limitations in current research regarding early identification (below 36 months) of the association between deficits in neurodevelopment and in the emotional-behavioural area become evident. Nevertheless, at older ages the empirical evidence is more solid. Thus, it is pertinent to analyse complex relationships in development during the first years of life. This knowledge can be crucial for detecting problems and developing prevention and early intervention programs.

### Aims and hypotheses

The overall aim of this work was to examine the relationship between the neurodevelopmental areas of the Bayley-III, with the possible existence of emotional/behavioral problems according to the CBCL 1.5–5 scales, in a sample of 231 boys and girls between 18 and 24 months.

In this study, the specific objectives were to: a) examine sex differences in areas assessed with the Bayley III scale and the CBCL 1.5–5 scale; b) establish the relationship between neurodevelopmental and emotional/behavioral areas and determine whether there are sex differences in that relationship; and finally, c) to verify how many children have low scores in neurodevelopment associated with high scores in emotional/behavioral problems.

Hypotheses were as follows: a) boys will present more difficulties than girls in areas of cognition, receptive and expressive communication, fine motor skills, and externalising behaviors; and girls will show more problems in gross motor areas and internalising behaviors; b) the lower the cognitive capacity, the lesser the communicative ability; and the lower the motor ability, the greater the inter and externalising problems; c) it is expected to find a minimum percentage of children who present deficits in any of the neurodevelopmental areas (cognitive, expressive communication, receptive communication, fine motor, gross motor) and also concurrent problems in the emotional-behavioural area.

## Materials and Methods

### Participants

The sample was recruited at the pediatric population of the university clinical hospital. Inclusion criteria for mothers were as follows: Caucasian and Spanish origin; 18–45 years of age; living in Health Area I (suburban and rural) or in certain districts of Health Areas VI and VII (mainly urban) of the Region of Murcia; planning to live in the area of study for at least 2 years; singleton pregnancy; non-assisted conception; intention to give birth at hospital; and normal echography findings at time of visit (no major malformations). Exclusion criteria included: chronic disease in the mother, pregnancy. Regarding criteria of babies, no medical pathology at birth and no serious or neurological disorders at 18 months.

The study began by asking 532 women who gave birth at the hospital for permission to test their babies at 18 months of age. Following this period, they were contacted for appointments, 188 did not wish to participate. Of the 344 mothers who agreed to participate, only 265 attended the evaluation session. The study included participation of 265 children who underwent the Bayley-III development test and mothers completed the CBCL 1.5–5 questionnaire. However, due to lack of information caused by loss of data in the CBCL, 34 children were excluded, with the final sample being 231 children of which 125 were boys and 106 girls (54.1% and 45.9%, respectively), with a mean age of 19.84 months (SD = 1.177). The minimum age was 17 months and maximum age 23 months, with 20 months the most frequent in the sample analysed. Table 1 summarizes the characteristics of the study population, while Table 2 shows age variable given by sex. This study was approved by the Ethics Committee of the HCUVA in Murcia.

**Table 1:** Central statistics for toal sample and by sex.


	BOYS	GIRLS	FULL SAMPLE

N (%)	125 (54.1)	106 (45.9)	231

Mean Age (S.D.)	19.84 (1.208)	19.83 (1.159)	19.84 (1.177)

Min–Max Age	18–23	17–23	17–23

Mo Age	20	20	20


Note. N = number of subjects, % = percentage, SD = Standar Desviation, Min = Minimun score, Max = Maximun score, Mo = Mode.

**Table 2:** Number and percentage of children by sex and age (in Months).


AGE	SEX. n (%)	

	BOYS	GIRLS

17 months	0 (0%)	1 (100%)

18 months	13 (59.1%)	9 (40.9%)

19 months	42 (57.5%)	31(42.5%)

20 months	40 (48.8%)	42 (51.2%)

21 months	15 (57.7%)	11 (42.3%)

22 months	13 (56.5%)	10 (43.5%)

23 months	2 (50%)	2 (50%)

Total	125	106


The mothers of the 231 children had a mean age of 33.3 years (SD = 4.7) (Min = 18; Max = 42 years). Fifty-nine point three percent had completed university studies, 31.6% had secondary education/non-university studies, and 9.1% had basic education. As regards their current occupation, 39% held management positions, 37.2% were skilled workers, and 3.9% were in unskilled jobs; 19.9% of mothers were not working at the time of the evaluation; 98% of women were married or in a relationship; 73.2% lived in the urban center, 10.8% in a residential area and 16% lived in a rural area.

### Instruments

As mentioned above, the evaluation instruments used in this project were the Bayley Scales of Infant and Toddler Development-III, for evaluation of children’s neurodevelopment between 15 days and 42 months of age and the Child Behavior Checklist (CBCL 1.5–5 years), to evaluate the presence of behavioural and/or emotional problems in children. The Bayley-III scale is divided into five scales: Cognitive, Language, Motor, Socio-Emotional and Adaptative domains (Bayley, 2006). For the purposes of the current study, only the cognitive, language, and motor items of Bayley Scales were administered to the child, since the Socio-Emotional and Adaptive scales are not validated in the Spanish version of Bayley (Bayley, 2006; Spanish adaptation CDIAP Parc Taulí, University of Murcia and the Pearson Clinical & Talent Assessment R&D Departament, 2015).

1. The cognitive scale (Raw score 0–91) evaluates visual preference, attention, memory, sensory motor, exploration and manipulation and concept formation, by computing a global score. In young children, how they explore new toys and activities, the way they solve problems (for example searching for a hindden object to evaluate the permanence of the object), and the ability to put together puzzles are evaluated.

2. The language scale measures a child’s ability to understand and utilize language, recognize objects, and follow directions. Thus, two subscales can be differentiated. The receptive subscale (verbal comprehension, vocabulary) assesses young children’s identification of images or objects, following simple directions, and interaction in routine play (for example, when faced with a picture with several drawings, they are asked “Where is the child’s car?”) (Raw score 0–49). And expressive communication subscale (babbling, gesturing, and utterances) in young children evaluates the use of words to name objects or images and to answer some questions (Raw score 0–48).

3. The motor scale is subdivided into two subscales. The gross motor subscale (sitting, standing, locomotion, and balance) in young children assesses the ability to crawl, make walking movements, support weight, stand, and walk without assistance (Raw score 0–72). And the fine motor subscale (grasping, perceptual-motor integration, motor planning, and speed) assesses young children’s ability to stack cubes, draw simple shapes, and manipulate small objects (Raw score 0–66).

The evaluation consists of the toddler, through a specific series of tasks coded as 0 if performed inadequately and as 1 if activity is performed adequately. This test offers scaled scores for each subscale (cognitive, receptive language, expressive language, fine motor and gross motor), and composite scores for three main scales. For this study, direct and scaled scores have been used. Clinical interpretation is based on scaled scores. The maximum score for each test is 19 and the minimum 1. The mean score is 10 and standard deviation 3, therefore toddlers with a score below 7 were considered to have significant delay.

On the CBCL scale (1.5–5 years) mothers individually complete 99 items with multiple response options (0 = not true (to my knowledge); 1 = somewhat or sometimes true; 2 = very true or often true). These items are grouped into 7 empirically based syndrome scales: emotional reactivity, anxiety/depression, somatic complaints, withdrawal, sleep problems, attention problems, and aggressive behavior. These seven scales are divided into two large groups of internalising and externalising symptoms, in addition to offering a total problem score. Likewise, scores obtained in the 7 scales are oriented toward the diagnostic criteria of the DSM-5 (APA, 2013).

Once questions are answered, a direct score is obtained which is transformed into a typical score according to the existing scales. These typical scores place the toddler in one of three possible ranges: nonclinical range (t = <64), borderline (t = 65–69), and clinical (t = >70). In the scale of internalising, externalising and total problems we would again have three ranges with changed t-scores: non-clinical range (t = <59), borderline range (t = 60–63) and clinical range (t = >64). Toddlers with a score equal to or above t = 65 seven scales and a score equal to or above t = 60 global scales were considered to have significant delay.

### Procedure

The present study is a descriptive cross-sectional study. The initial sample of children was randomly selected from infants born between 2015 and 2017, at the university hospital “Virgen de la Arrixaca” (HCUVA) in Murcia (Spain). Participants were selected from the Pediatric Service, where mothers, voluntarily confirming their participation in the study, provided informed consent authorizing the research team to collect data related to their child. When children turned 18 months old, the Pediatric Psychology Unit of the HCUVA performed the neurodevelopmental test. Parents gave further consent authorizing the test and subsequent use of data obtained for research. After parents received explanation of what the session would consist of, a qualified professional trained in the Bayley-III test carried out evaluation of the child. Meanwhile, parents completed the CBCL questionnaire. Both parents were requested to complete the CBCL separately in order to have a broader vision of the child’s problems, but only mothers attended the session. Both tests were applied in one session. Duration of tests was usually two hours and normally requires a single evaluation session. When toddlers were reluctant to finish or were tired (15%), they were evaluated again the following day to ensure reliability of data collected.

### Data Analyses

For data processing, the statistical program SPSS v.27.0 was used. Quantitative variables were described using means and standard deviations, and qualitative variables using percentages. Comparisons of the quantitative variables between two groups were made using the Student’s t-test for independent samples once assumptions of normality (Kolmogorov-Smirnov test) and homogeneity of variances (Levene’s test) had been verified. Complementarily, comparisons were evaluated using the Bayesian t test. The interpretation of the Bayes factor was performed according to the classification of Wagenmakers et al. (2011), where values of 1 and 3 are interpreted as anecdotal evidence towards null hypothesis (BF01), between 3 and 10 as substantial evidence, between 10 and 30 strong evidence, while between 30 and 100 very strong evidence. For comparisons between groups of qualitative variables, the Chi-square test or Fisher’s exact test was used. Correlations between variables were studied using the Pearson correlation coefficient. Multiple linear regression models were performed introducing the dimensions of the CBCL scale as dependent variables and the Bayley scale dimensions as independent variables and sex as the moderator variable. The effect size for the student t-tests was measured using Cohen’s d, considering that values less than 0.2 indicate a small effect size, 0.5 of medium magnitude, and 0.8 indicates a high magnitude effect (Cohen, 1988). For the Chi-square or Fisher tests, the effect size was measured using Cramer’s V, considering that values of 0.10–0.29 indicate a low effect, 0.30–0.49 indicate a medium effect, and values from 0.50 as high. A significance level of 5% (p < 0.05) was adopted.

## Results

### Sex differences

The first aim was to examine the influence of sex in the areas of neurodevelopment and emotional-behavioural, through the *t*-Student test. Table 3 shows the difference of mean scores on Bayley/CBCL scales and Bayesian analysis according to sex. As regards the Bayley scale, statistically significant differences were found based on sex in the areas of receptive communication (*t* (229) = 2.24; *p* = 0.026) and expressive communication (*t* (229) = 3.34; *p* = 0.001). Girls obtained a higher mean score than boys in both areas: in receptive communication (*M* = 24.02; *SD* = 3.75 *vs M* = 22.83; *SD* = 4.22), with a small effect size (*d* = 0.30), and in expressive communication (*M* = 25.25; *SD* = 3.83 vs *M* = 23.45; *SD* = 4.31), it was also small-medium (*d* = 0.44). The Bayes factor in the area of receptive communication (*BF*_01_ = 0.86) indicated anecdotal evidence in favour of the alternative hypothesis, which in the 95% credibility interval was between –2.22 and –0.15. For expressive communication (*BF*_01_ = 0.05), the evidence was strong for the alternative hypothesis (credibility interval –2.87 to –0.75). In the remaining areas (cognitive, fine motor and gross motor) no statistically significant differences were found according to sex, with effect sizes less than 0.20, and substantial evidence in favor of the null hypothesis in the Bayesian analysis. In the emotional-behavioural area, statistically significant differences by sex were found only in the withdrawal syndrome ((*t* (229) = 2.76; *p* = 0.006). The mean scores for girls were higher than for boys (*M* = 1.24; *SD* = 1.70 vs *M* = 0.68; *SD* = 1.33), with a small effect size (*d* = 0.37). The Bayes factor (*BF*_01_ = 0.25) indicated substantial evidence for the alternative hypothesis, which in the 95% credibility interval was found between –0.96 and –0.16. There were no statistically significant differences between boys and girls in the rest of the areas. Effect sizes were less than 0.20, and the evidence was substantial in favour of the null hypothesis in the Bayesian analysis (Table 3).

**Table 3:** Difference of means (Student-t), effect size and Bayesian analysis according to sex in the BAYLEY/CBCL scales.


BAYLEY	SEX. MEAN (*SD*)				BAYESIAN T TESTS EVALUATING		
	
	GIRLS	BOYS	*P*-VALUE	*D*	BAYES FACTOR (*BF0*_1_)	95% CREDIBLE INTERVAL	EVIDENCE CATEGORY (IN FAVOR OF *H*)

Cognitive	61.73 (6.23)	60.86 (6.45)	0.3	0.14	5.72	–2.52; 0.78	Substantial H_0_

Receptive communication	24.02 (3.75)	22.83 (4.22)	0.026	0.30	0.86	–2.22; –0.15	AnecdoticH_1_

Expressive Communication	25.25 (3.83)	23.45 (4.31)	0.001	0.44	0.05	–2.87; –0.75	Strong H_1_

Fine motor	37.98 (3.05)	37.43 (2.71)	0.149	0.19	3.50	–1.31; 0.21	Substantial H_0_

Gross motor	53.22 (2.59)	52.68 (2.91)	0.143	0.19	3.39	–1.25; 0.18	Substantial H_0_

CBCL							

Emotional reactivity	1.52 (1.62)	1.59 (1.97)	0.753	–0.04	9.20	–0.39; 0.54	Substantial H_0_

Anxiety/depression	2.15 (1.70)	1.85 (1.57)	0.164	0.18	3.76	–0.73; 0.13	Substantial H_0_

Somatic complaints	1.16 (1.46)	1.17 (1.56)	0.961	–0.01	9.64	–0.38; 0.4	Substancial H_0_

Withdrawal	1.24 (1.70)	0.68 (1.33)	0.006	0.37	0.25	–0.96; –0.16	Substancial H_1_

Sleep problems	3.53 (2.83)	3.40 (2.70)	0.719	0.05	9.06	–0.85; 0.59	Substancial H_0_

Attention problems	2.77 (1.67)	2.60 (1.63)	0.453	0.10	7.33	–0.59; 0.27	Substancial H_0_

Agressiveness	9.83 (4.96)	9.14 (5.25)	0.306	0.14	5.79	–2.02; 0.63	Substancial H_0_

Internalising	6.08 (4.60)	5.31 (4.82)	0.217	0.12	4.60	–1.99; 0.46	Substancial H_0_

Externalising	12.63 (6.15)	11.73 (6.38)	0.274	0.16	5.39	–2.53; 0.72	Substancial H_0_

Total problems	29.26 (14.81)	26.97 (15.06)	0.246	0.15	5.01	–6.17; 1.59	Substancial H_0_


Note. *d* = Cohens’s *d*, H_0_ = Null hypothesis, H_1_ = Alternative hypothesis.

Differences based on sex in participants with or without significant problems were verified, taking into account the cut-off point of scales to determine subjects with significant delay (Table 4). First, the number of children who presented a significant delay for each score on the Bayley and CBCL scales was identified. Next, the percentage for boys and girls was analyzed, and finally it was verified wheter there were statistically significant differences between both sexes. The Chi square test and Fisher’s exact test were performed. Results indicated the influence of sex was only significant for expressive communication (*p* = 0.018), 80% of boys had problems versus 20% of girls, with a small effect size (Cramer’s *V* = 0.16). No association was found between sex and the rest of the neurodevelopmental areas or emotional-behavioural areas.

**Table 4:** Number and percentage of children by sex and in the total sample presenting a significant delay for the different scores of the BaAYLEY and the CBCL scales.


	SEX. n (%)		*P-VALUE*	*FULL SAMPLE n(%)*

	GIRLS	BOYS		

BAYLEY				

Cognitive^1^	2 (50)	2 (50)	1	4 (1.7)

Receptive communication	7 (46.7)	8 (53.3)	0.95	15 (6.5)

Expressive communication^1^	4 (20)	16 (80)	0.018*	20 (8.7)

Fine motor^1^	1 (50)	1 (50)	1	2 (0.9)

Gross motor^1^	0 (0)	1 (100)	1	1 (0.4)

CBCL				

Emotional reactivity	6 (54.5)	5 (45.5)	0.555	11 (4.8)

Anxiety/depression^1^	1 (50)	1 (50)	1	2 (0.9)

Somatic complaints^1^	3 (50)	3 (50)	1	6 (2.6)

Withdrawal^1^	3 (33.3)	6 (66.7)	0.513	9 (3.9)

Sleep problems	7 (31.8)	15 (68.2)	0.164	22 (9.5)

Attention problems^1^	5 (50)	5 (50)	1	10 (4.3)

Agressiveness^1^	3 (60)	2 (40)	0.663	5 (2.2)

Internalising	8 (47.1)	9 (52.9)	0.92	17 (7.4)

Externalising	11 (44)	14 (56)	0.841	25 (10.8)

Total problems	8 (50)	8 (50)	0.732	16 (6.9)


^1^ Fisher’s exact test; *p < 0.05.

### Association between Bayley and CBCL scales

As a second aim, the relationship between the areas of neurodevelopment and the problems in the emotional-behaviuoral areas were analysed (Table 5). It was also verified whether sex moderated this relationship. In the study of the relationship between areas of neurodevelopment and problems in the emotional-behavioural areas, significant negative sign correlations were found, thus low scores in neurodevelopment were related to high scores in emotional-behavioural problems. All presented a small effect size (from *r* = –0.136 in the relationship between receptive communication and aggressiveness to *r* = –0.257 in the relationship between expressive communication and withdrawal). Receptive communication had the highest number of significant relationships with CBCL (total problems, internalising, externalising, and withdrawal syndromes, attention problems, and aggressiveness). The cognitive area was significantly related to the total, internalising, and externalising scales, as well as anxiety/depression syndromes, withdrawal, and attention problems. Expressive communication was related to total problems, internalising problems, and emotional reactivity and withdrawal syndromes. In the motor area, a significant relationship was only found between fine motor skills and anxiety/depression and withdrawal syndromes.

**Table 5:** Correlations between BAYLEY subscales and specific and general CBCL subscales.


CBCL	BAYLEY				

	COGNITIVE	RECEPTIVE COMMUNICATION	EXPRESSIVE COMMUNICATION	FINE MOTOR	GROSS MOTOR

Emotional reactivity	–0.057	–0.044	–0.154*	–0.059	–0.06

Anxiety/depression	–0.150*	–0.093	–0.097	–0.162*	–0.113

Somatic complaints	–0.039	–0.077	–0.023	0.004	0.011

Withdrawal	–0.161*	–0.242**	–0.257**	–0.144*	–0.053

Sleep problems	0.063	–0.012	–0.013	0.045	–0.082

Attention problems	–0.166*	–0.177**	–0.064	–0.086	–0.062

Agressiveness	–0.154*	–0.136*	–0.096	–0.096	–0.051

Internalising	–0.137*	–0.155*	–0.184**	–0.125	–0.077

Externalising	–0.157*	–0.160*	–0.088	–0.096	–0.057

Total problems	–0.143*	–0.144*	–0.159*	–0.111	–0.074


*p < 0.05 **p < 0.01.

After the correlation analyses, multiple linear regression models were performed, introducing the dimensions of the CBCL scale as dependent variables, the dimensions of the Bayley scale as independent variables, and sex as the moderator variable. None of the models was statistically significant.

### Sample comorbidity

Participants included in the sample were categorized into two levels (no-yes), according to the cut-off point of the two scales. On the Bayley scale to know those children who had an adequate level of development (scale score ≥7) or presented difficulties (scale score <7), and on the CBCL scale those who did not present problems (typical score <60 for the general and <65 for syndromes) and those who had significant emotional-behavioural problems (≥60 and ≥65, respectively) (Table 4). On the Bayley scale, the most prevalent area was expressive communication in 8.7% of the sample (20 subjects), followed by receptive communication in 6.5% (15 subjects). Only one subject presented problems in the gross motor area. Regarding the CBCL, 6.9% of study participants presented problems on the total emotional-behavioural scale. A greater number of subjects with externalising problems (10.8%) compared to internalising (7.4%) were found. The most prevalent syndrome was sleep problems (9.5%), followed by emotional reactivity (4.8%). The least prevalent was anxiety/depression (0.9%).

The aim of the study was to learn the number of participants who simultaneously presented difficulties in the neurodevelopmental and emotional-behavioral areas. In Table 6 the five Bayley subscales and the seven specific subscales of CBCL are presented. Of the 231 participants evaluated, 155 (67.1% of the sample) did not present difficulties. Of the 76 subjects (32.9%) who had some problem in either area (Bayley-CBCL), 67.10% of these obtained scores above the cut-off point on CBCL subscales, and 23.68% did so on the Bayley scale, with 9.21% (7 subjects) showing at least one problem in each area (neurodevelopment/emotional-behavioural).

**Table 6:** Number of participants presenting simultaneous problems on the BAYLEY and CBCL scales.


		CBCL		TOTAL

		NO PROBLEMS	PROBLEMS	

BAYLEY	No problems	155	51	206

	Problems	18	7	25

Total		173	76	231


## Discussion and Conclusions

The general aim of this study was to examine the relationship between the neurodevelopmental areas of the Bayley-III, with the possible existence of emotional-behavioural problems according to the CBCL 1.5–5, in a sample of 231 boys and girls with typical development and a mean age of 19.84 months. A series of specific objectives and their corresponding hypotheses were established based on the scientific literature.

The first aim involved analysing the influence of sex in the neurodevelopmental and emotional/behavioural areas. Findings provide evidence of better language skills in girls in both language areas. In receptive communication the effect size was small, Bayes analysis indicated anecdotal evidence in favour of the alternative hypothesis. In expressive communication, the effect size was small-moderate, with strong evidence for the alternative hypothesis, based on Bayesian analysis. Regarding the emotional/behavioural area, girls presented more withdrawal problems, with a small effect size, and substantial evidence for the alternative hypothesis according to Bayes. No statistically significant differences were found in the rest of areas evaluated, with substantial evidence in favour of the null hypothesis. In contrast, when prevalence rates of participants with and without deficits in each area were compared, only statistical significance was found in expressive communication, with greater difficulties being found in boys. Thus, these results only partially support our hypothesis. They are consistent with works which evaluate the language area (Henrichs et al., 2013; Koutra et al., 2012; Krog & Væver, 2019; Lung et al., 2009; Rautakoski et al., 2021; Suhonen et al., 2018). In this regard, this study shows that girls obtain an advantage with strong evidence in the area of expressive communication. Some possible explanations for sex differences found in the Bayley-III might indicate either real differences in the test measurement or differences owing to how boys and girls respond to an exam situation (Krog & Væver, 2019). Likewise, concerning the emotional-behavioural area, our results could indicate a tendency to develop internalising problems in young girls (withdrawal problems), as confirmed by research in preschool and school samples (Marković et al., 2016; Pourhoussein et al., 2015; Shaikh & Shinde, 2018).

The second aim was to establish the relationship between areas of neurodevelopment and problems in the emotional-behavioural area. It was also verified whether sex moderated this relationship. A significant negative relationship was confirmed, meaning that fewer neurodevelopmental deficits which although small meant more difficulties in emotional-behavioural problems. Receptive communication had the highest number of significant relationships with CBCL (total problems, internalising, externalising, and withdrawal syndromes, attention problems, and aggressiveness). The cognitive area was significantly related to the total, internalising, and externalising scales, as well as anxiety/depression syndromes, withdrawal, and attention problems. Expressive communication was related to total problems, internalising problems, and emotional reactivity and withdrawal syndromes. In the motor area, a significant relationship was only found between fine motor skills and anxiety/depression and withdrawal syndromes. Multiple linear regression models did not confirm the predictive effect of any neurodevelopmental area on emotional-behavioural problems, nor did sex moderate these relationships in this sample.

Therefore, the proposed hypothesis was only partially confirmed, since in cognitive and receptive communication areas, low-magnitude negative associations were obtained with internalising and externalising, coinciding with some authors who have obtained similar results in these development stages (Conway et al., 2017; Flouri et al., 2018; Morin et al., 2017; Thurm et al., 2018). Notwithstanding, it should be noted that some researchers have also more clearly observed a unidirectional relationship between deficits in early receptive language as a predictor of later internalising behaviour problems in samples of pre-schoolers, and also suggest some associations would be moderated by sex (e.g., Bichay-Awadalla, et al., 2020; Bornstein et al., 2013). However, various studies with samples of preschool and school age children provide evidence of the bidirectional relationship between language skills and emotional-behavioural problems (Bichay-Awadalla et al., 2020; Bornstein et al., 2013), although this pattern has not yet been confirmed in children under 36 months of age (Thurm et al. 2018). It is therefore essential to examine both areas of language (expressive and receptive), as indicated by Chow et al. (2018) in their review of preschool and school samples, as these can give rise to different patterns of association with emotional-behavioural disorders, children with low receptive skills being more likely to present behavioural alterations. In this study, low levels of expressive communication were linked to internalising problems, and low levels of receptive communication with both inter and externalising problems.

The third aim of the study focused on learning the number of participants who simultaneously presented difficulties in neurodevelopmental and emotional-behavioural areas. The largest prevalence rate in neurodevelopment was found in expressive communication (8.7%). In the emotional-behavioural area, more children presented externalising problems (10.8%), with a total of 6.9% problems, including both clinical and subclinical cases. 32.9% of the sample presented at least deficits in one of the Bayley or CBCL areas. Of these, only 9.21% (7 participants) showed at least one deficit in each area (neurodevelopmental and emotional-behavioural) and greater comorbidity was observed in boys.

Thus, the proposed hypothesis is confirmed. The prevalence rates of neurodevelopmental deficits were lower than those obtained in the studies reviewed in the community population (Ballot et al., 2017; Cromwell et al., 2014). Our findings in the emotional-behavioural area were not consistent with those found in other studies.

In general, the literature has documented that if the onset of symptoms occurs at an early age (3 years and older), both in the neurodevelopmental and emotional-behavioural areas, and there is a concurrence of internalising symptoms, externalising symptoms, and language ability, this might also indicate that common risk factors converge in early stages of life (Tamayo et al., 2023). Nevertheless, to the best of our knowledge, this remains to be researched in children under three years of age. Hence the importance of continuing to investigate these relationships in this early period of development, which justifies this study.

### Strengths and limitations

The most notable strength of this study is it provides evidence to the scarce existing literature on the relationship between the various areas of child development, from which relevant practical implications are derived. Moreover, a sufficiently large sample is analysed from a typical community population and covers the period prior to the preschool stage. Two universally well-established and validated assessment instruments have been used. In addition, the use of Bayesian statistics, a method used in other disciplines but innovative in psychology, enabled us to examine the influence of sex in areas of child development, unlike most existing studies that have exclusively used frequentist approaches.

Some potential limitations should also be considered, including the cross-sectional study design, thus not considering possible longer-term associations and likely changes; similarly, it would be necessary to consider drawbacks inherent in evaluation methods (use of a single source to collect information on each area); other than sex, potential confounding variables (biological and environmental) have not been examined; only the unidirectional relationship of the influence of neurodevelopment in the emotional-behavioural area has been analysed and not vice versa.

It is worth noting the difficulty when comparing our findings with data obtained in previous studies, due to the heterogeneity of results observed, likely because of variability in methodology used (size and type of sample, age of participants, evaluation tools used and statistical analysis).

### Future lines

The cross-sectional results obtained in this work must be confirmed. Therefore, it is necessary to broaden and deepen research on the relationship between neurodevelopment and the emotional-behavioural area. These should both be analysed as important indicators of development in unison and in both directions, rather than being considered as independent indicators (Bichay-Awadalla et al., 2020; Chow et al., 2018; Tamayo et al., 2023), not only in school or later stages, but also at early ages. Furthermore, these studies should be performed longitudinally to verify possible trajectories and combinations throughout development (Lu et al., 2020). It would be advisable to collect information from additional sources and measurement methods, as good evaluation practices require (Chow & Hollo, 2018; Chow et al., 2018; Miller et al., 2018). Future studies should analyse the possible moderating influence of additional biological (gestational age, birth weight, etc.) and contextual (parental age, quality of education, stress, etc.), as well as the underlying explanatory mechanisms (Clegg et al., 2015; Conway et al., 2017; Robert et al., 2018; Thurm et al., 2018). Finally, it is the relevance of examining the socio-emotional competencies of younger children and their relationship with neurodevelopment, which can function as critical early correlates of future problematic developments in the emotional-behavioural area (Horwitz et al., 2003; Rautakoski et al., 2021).

### Practical implications

Relationships analysed in the present study have significant implications for clinical and educational practice.

Early identification of neurodevelopmental deficits and emotional-behavioural problems is a priority and can contribute to early diagnosis in these areas. Otherwise, the life quality of children and their families can be affected (Maggio et al., 2014). Therefore, it is essential that professionals in the educational and health fields recognise intersections between neurodevelopment and the emotional-behavioural area (Chow & Hollo, 2022; Thurm et al., 2018). The detection of deficits in one domain should lead to the evaluation of the other (Tamayo et al., 2023), in order for these to be referred to specific early intervention programs (Conway et al., 2017). The emotional and behavioural problems which parents of young children easily identify are the gateway to seeking professional help. This would be an opportunity for evaluation of communicative and cognitive areas of neurodevelopment, and identification of possible deficits. Furthermore, professionals who perform specific intervention programs in the cognitive or linguistic areas should bear in mind the importance of working with the family to promote healthy emotional development in their children, in order to prevent future alterations in this area.

Early awareness of these relationships early would make it possible to intensify preventive efforts and/or more intensive interventions, which could promote a typical developmental trajectory, thus saving many personal, family, school, and financial costs.

### Conclusions

Our findings showed that differences between boys and girls were small, with the latter performing slightly better overall. Girls possess better language skills, finding greater evidence of this advantage in expressive language. Boys presented clinical scores in expressive communication. Likewise, girls scored higher in internalising problems, such as social withdrawal. The predictive value of any neurodevelopmental area on emotional-behavioural problems was not confirmed, nor did sex moderate these relationships. Children who had low cognitive abilities and those who presented low receptive communication showed more inter and externalising difficulties, with a low intensity relationship. In this sample, the number of participants who simultaneously manifested significant deficits in both domains (neurodevelopmental and emotional-behavioural) was very reduced.

Future research should corroborate these results and the characteristics of the relationship found at these early ages. Detecting the population at risk in the first two years of life would allow implementation of interventions aimed at improving neurodevelopmental deficits and emotional-behavioural problems. Promoting these actions could optimise the effectiveness of subsequent outcomes and long-term quality of life.

## Data Accessibility statement

The original data used in this study were obtained from the research Project “Psychological Intervention in Children with Health Problems”, conducted by the Research Group on Clinical Child and Adolescent Psychology (E069-03) at the University of Murcia, under the direction of Dra. Concepción López Soler. This project was funded by the Foundation for Health Research and Training of the CARM. Due to confidentiality agreements established with the participants and the research ethics to preserve the integrity of the ongoing nature of the research, sharing the original data is not possible for the time being.
